# BARRIERS TO REDUCING AND PREVENTING INVOLUNTARY TREATMENT: THE PERSPECTIVE OF PROFESSIONAL CAREGIVERS

**DOI:** 10.1093/geroni/igac059.1670

**Published:** 2022-12-20

**Authors:** Michel Bleijlevens, Jules Willems, Valeria Lima Passos, Jan Hamers

**Affiliations:** Maastricht University, Maastricht, Limburg, Netherlands; Maastricht University, Maastricht, Limburg, Netherlands; Maastricht University, Maastricht, Limburg, Netherlands; Maastricht University, Maastricht, Limburg, Netherlands

## Abstract

Measures to which a person resits and/or does not provide consent for are defined as involuntary treatment. The use of involuntary treatment violates the autonomy of (older) persons and causes more harm than benefit. Moreover, it contradicts the values of person-centred care. Nevertheless, its use among Persons Living with Dementia (PLWD) is still common practice and remains difficult to prevent and/or reduce. The aim of this study was to gain insights into the barriers towards the prevention and/or reduction of involuntary treatment in long-term geriatric care. We conducted a cross-sectional, mixed-methods study, including an online survey for professional caregivers, and a semi-structured focus group interview with professional caregivers. A total of 218 participants completed the questionnaire. The percentage of participants that experienced barriers in one of the twenty-two survey items ranged from 15% to 42%. Lack of time; the experienced need to use involuntary treatment; uncertainty about responsibilities of stakeholders; and a lack of knowledge on methods to prevent and/or reduce its use were most seen as barriers. Nursing staff experienced a lack of time more often than other professional caregivers. Working in home care and having no former experience with involuntary treatment usage increased perceived barriers. Participants of the focus group interview confirmed these findings. One out of four professional caregivers experience barriers hindering prevention and/or reduction of involuntary treatment. More research is needed to gain better understanding on how professional caregivers can be supported aiming to, remove barriers and consequently prevent and/or reduce the use of involuntary treatment.

